# The endothelial protein C receptor rs867186-GG genotype is associated with increased soluble EPCR and could mediate protection against severe malaria

**DOI:** 10.1038/srep27084

**Published:** 2016-06-03

**Authors:** Estela Shabani, Robert O. Opoka, Paul Bangirana, Gregory S. Park, Gregory M. Vercellotti, Weihua Guan, James S. Hodges, Thomas Lavstsen, Chandy C. John

**Affiliations:** 1 Department of Pediatrics, Division of Global Pediatrics, University of Minnesota, 2001 6^th^ Street SE, Minneapolis, MN, 55455, USA; 2 Ryan White Center for Pediatric Infectious Disease and Global Health, Department of Pediatrics, Indiana University School of Medicine, 1044 West Walnut Street, Indianapolis, IN, 46202, USA; 3 Department of Pediatrics and Child Health, Makerere University School of Medicine, PO Box 7072, Kampala, Uganda; 4 Department of Psychiatry, Makerere University School of Medicine, PO Box 7072, Kampala, Uganda; 5 Department of Medicine, University of Minnesota Medical School, 401 East River Parkway, Minneapolis, MN, 55455, USA; 6 Division of Biostatistics, University of Minnesota School of Public Health, 420 Delaware St SE, Minneapolis, MN, 55455, USA; 7 Centre for Medical Parasitology, Department of International Health, Immunology & Microbiology, University of Copenhagen and Department of Infectious Diseases, Rigshospitalet, Tagensvej 20, 2200, Copenhagen, Denmark

## Abstract

The endothelial protein C receptor (EPCR) appears to play an important role in *Plasmodium falciparum* endothelial cell binding in severe malaria (SM). Despite consistent findings of elevated soluble EPCR (sEPCR) in other infectious diseases, field studies to date have provided conflicting data about the role of EPCR in SM. To better define this role, we performed genotyping for the rs867186-G variant, associated with increased sEPCR levels, and measured sEPCR levels in two prospective studies of Ugandan children designed to understand immunologic and genetic factors associated with neurocognitive deficits in SM including 551 SM children, 71 uncomplicated malaria (UM) and 172 healthy community children (CC). The rs867186-GG genotype was more frequent in CC (4.1%) than SM (0.6%, *P* = 0.002). The rs867186-G variant was associated with increased sEPCR levels and sEPCR was lower in children with SM than CC (*P* < 0.001). Among SM children, those who had a second SM episode showed a trend toward lower plasma sEPCR both at initial admission and at 6-month follow-up compared to those without repeated SM (*P* = 0.06 for both). The study findings support a role for sEPCR in severe malaria pathogenesis and emphasize a distinct role of sEPCR in malaria as compared to other infectious diseases.

Cerebral malaria (CM) and severe malarial anemia (SMA) remain leading causes of morbidity and mortality from *Plasmodium falciparum* infection. CM has a mortality rate of 13–15%^1^,^2^,^3^,^4^ and survivors of CM are at high risk of short-5 and long-term^6^ neurocognitive impairment. SMA has a substantial burden in Sub-Saharan Africa causing 20% of *P. falciparum* hospitalizations^7^. Little is known about why only certain children develop severe malaria (SM) and why some children with SM have worse clinical outcomes.

Binding of infected erythrocytes (IEs) to host endothelium via *P. falciparum* erythrocyte membrane protein-1 (PfEMP-1) is an important driver of CM as it prevents parasite clearance, and is associated with increased local vasoconstriction, hypoxia and acidosis^2^,^8^,^9^,^10^,^11^,^12^. Binding of IEs and sequestration are also important in SMA^13^,^14^ together with other factors such as erythrocyte lysis and suppression of hematopoiesis^15^. PfEMP-1 can bind to various host-receptors^16^,^17^ and recently, PfEMP-1 variants associated with severe malaria^18^,^19^,^20^ were shown to bind EPCR^21^, suggesting an important role for this receptor in pathogenesis of severe malaria.

EPCR regulates coagulation by enhancing activation of protein C (PC)^22^,^23^,^24^, and has cytoprotective functions when bound to activated PC (aPC)^25^. EPCR is cleaved into its soluble form (sEPCR) by tumor necrosis factor-α converting enzyme (TACE)^26^. TACE’s activity is increased, by TNF-α, IL-1β and thrombin generation^27^. EPCR gene (*PROCR*) variations can also affect sEPCR levels. The rs867186-G variant in exon 4 of *PROCR* causes a serine-to-glycine substitution in the transmembrane region, making bound EPCR more susceptible to shedding^28^,^29^.

The evidence that PfEMP-1 binds to EPCR at the binding site of PC and aPC^21^,^30^, reducing the production and cytoprotective effects of aPC^31^ makes EPCR a potential important link between sequestration, coagulation defects and endothelial activation in severe malaria. sEPCR can bind to IEs and inhibit their adhesion to human brain microvasculature endothelial cells^31^. Reduced EPCR was observed in autopsy samples from pediatric CM patients, which coincided with sequestration of IEs and fibrin accumulation^32^. Also, a study from Thailand found that rs867186-GG genotype was protective against severe malaria^33^.

However, other studies, including studies in African children showing no association between the rs867186-G variant and severe disease^34^,^35^ and conflicting studies showing an increase^36^ or decrease^35^ in sEPCR levels in severe malaria suggest that the contributions of the rs867186-GG genotype and sEPCR levels in severe malaria are still unclear. These unresolved questions about the association of severe malaria with the rs867186-G variant and changes in sEPCR levels led us to investigate these associations in a cohort of Ugandan children with severe malaria (cerebral malaria or severe malarial anemia), uncomplicated malaria, and otherwise healthy Ugandan children.

## Results

### Baseline characteristics

**Figure 1:**
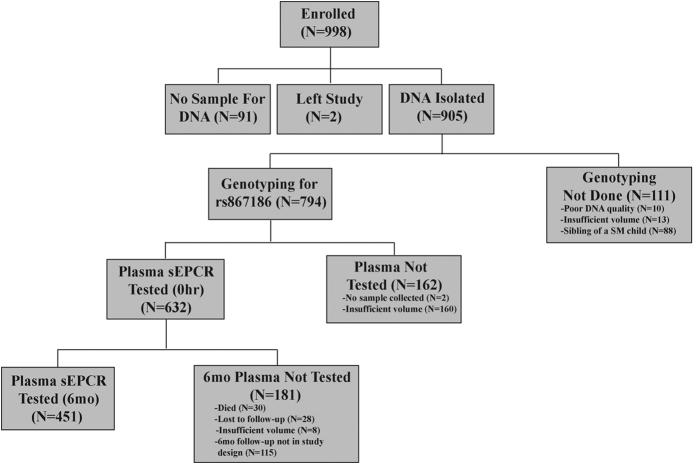
Study profile. Abbreviations: sEPCR, soluble endothelial protein C receptor; SM, severe malaria.

Of the 794 children who were genotyped for rs867186, sEPCR levels at enrollment were quantified in 484 SM (277 CM and 207 SMA), 38 UM and 110 CC (see Methods, Fig. 1). Children with severe malaria were younger than children with UM or CC (Table 1). sEPCR level was associated with age in children with SM (Spearman’s rho −0.10, *P* = 0.03) but not in children with UM or healthy controls (*P* > 0.64 for all). sEPCR level was not associated with sex in any group (*P* > 0.17 for all).

### Prevalence of rs867186-G EPCR variant in children with severe malaria, uncomplicated malaria and healthy community children

The prevalence of rs867186-G was higher in healthy controls than severe malaria children in an additive model (*P* = 0.006, Table 2). A recessive model looking at the prevalence of GG genotype vs. AG + AA showed that healthy community children had a higher prevalence of the GG genotype (4.1%) compared to children with SM (0.6%, *P* = 0.002). The GG genotype was associated with an 87% reduced rate of severe malaria (odds ratio (OR) 0.13, 95% CI 0.03–0.50, *P* = 0.003). The prevalence of AA vs. GG + AG did not differ significantly between the disease groups and CC in a dominant model (*P* > 0.37 Table 2).

The rs867186-G variant tags haplotype 3 of *PROCR.* We also assessed the prevalence of haplotype 1, tagged by rs9574-C, as it has been associated with increased risk of thromboembolism in some^37^ but not all^38^ studies, and one study associated the presence of both these haplotypes with protection from severe sepsis^39^. In our cohort, the prevalence of rs9574-C did not differ significantly between malaria groups and CC under a recessive, dominant or additive model (*P* > 0.13 for all comparisons, Supplemental Table 1). Moreover, children who had both variants were not less likely to have severe malaria (*P* > 0.98, Supplemental Table 2).

### Levels of soluble EPCR in children with severe malaria were lower at enrollment but normal at six-months follow-up

**Figure 2:**
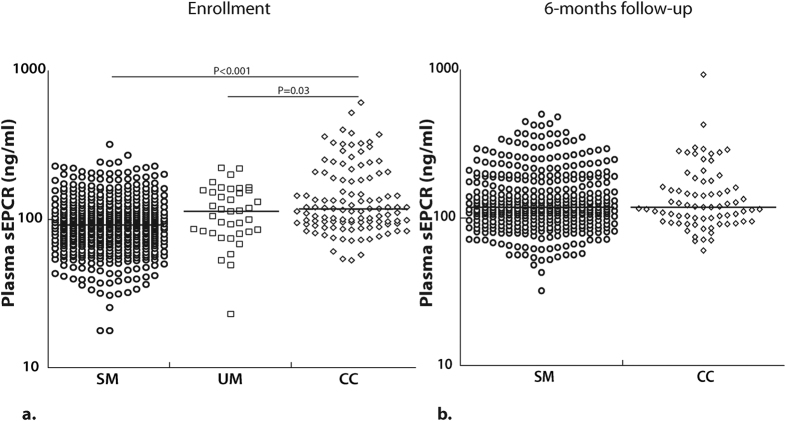
Plasma sEPCR levels are lower with increased disease severity at enrollment, but normal at 6-months follow-up. (**a**) sEPCR levels (on a logarithmic scale) at enrollment and (**b**) at 6-months follow-up. The horizontal line represents median values. Severe malaria (SM), uncomplicated malaria (UM), community controls (CC).

Plasma sEPCR levels at enrollment were significantly lower in children with SM (n = 484, median, ng/ml [25^th^ percentile, 75^th^ percentile], 91.8 [69.4, 118]) compared to CC (n = 110, 117 [94.9, 189], *P* < 0.001, Fig. 2a). sEPCR levels in children with uncomplicated malaria (UM, n = 38, 114 [82.4, 156]) were lower than CC (*P* = 0.03), and higher than children with SM (Fig. 2a), but the latter comparison did not reach statistical significance (*P* = 0.07) likely because of the small sample size of children with UM. When controlling for age, the (log-transformed) sEPCR level was significantly lower in the SM group compared to CC (*P* < 0.001). The difference between the UM and CC group was modest (p = 0.055).

At six months post-discharge, sEPCR levels in children with SM (n = 378, 118 [94.7, 176]) did not differ significantly from CC (n = 73, 118 [94.8, 163], *P* = 0.77, Fig. 2b), and were similar to the CC levels at enrollment. These results suggest that lower plasma sEPCR levels in children with SM occur most notably during the disease processes of severe malaria.

### Plasma sEPCR levels at enrollment and 6-month follow-up trend lower in children with repeated SM

**Figure 3:**
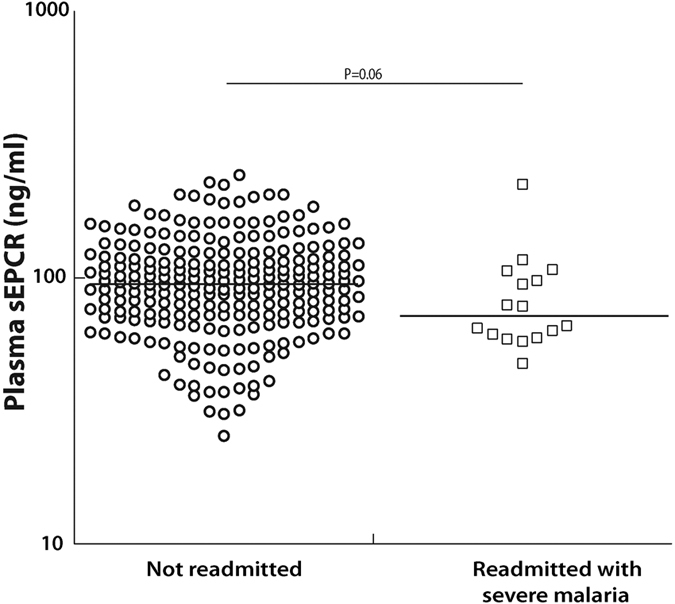
Plasma sEPCR levels were lower in the children readmitted with severe malaria. sEPCR levels (on a logarithmic scale) at enrollment in children with SM separated by whether they were readmitted within 6-mo of discharge for severe malaria. The horizontal line represents median values.

Readmission rates for severe malaria were assessed in the children from the CM/SMA study who were not enrolled in a subsequent nested study of iron treatment and who did not leave the study (301 children: CM, n = 156, SMA, n = 145), as iron could change risk of readmission. We compared plasma sEPCR levels at enrollment in children with severe malaria that were readmitted with severe malaria within 6-months of discharge versus sEPCR levels in children that were not readmitted with severe malaria. sEPCR levels at enrollment were lower in children who were readmitted with severe malaria as compared to not readmitted (readmitted with severe malaria within 6-months of discharge n = 16, median, [25^th^ percentile, 75^th^ percentile] ng/ml, 72.2 [60.5, 102] vs. not readmitted n = 244, 95.0 [72.8, 123], *P* = 0.06, Fig. 3). Readmitted children also had lower sEPCR levels at 6-month follow-up (readmitted n = 15, median, [25^th^ percentile, 75^th^ percentile] ng/ml, 101 [87.6, 116] vs. not readmitted n = 226, 121 [94.1, 176], *P* = 0.06).

### Association of rs867186-G variant with higher sEPCR levels

**Figure 4:**
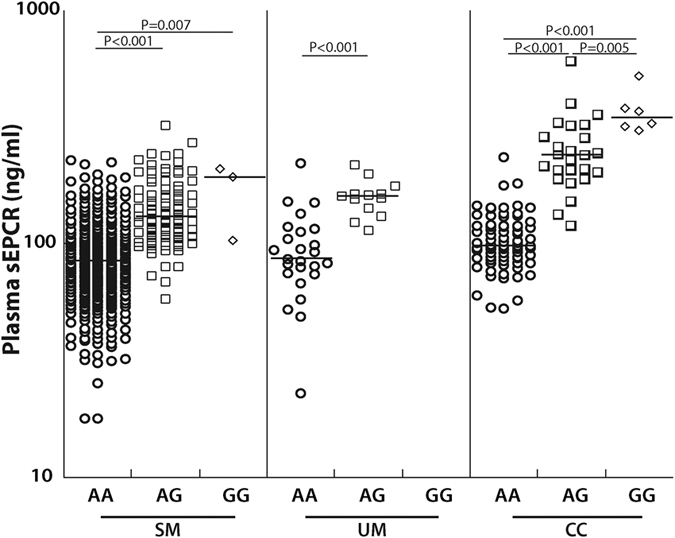
rs867186-G is associated with higher sEPCR level. sEPCR levels are represented on a logarithmic scale and each disease group is separated by rs867186 genotype: AA, AG or GG. The horizontal line represents median values. Severe malaria (SM), uncomplicated malaria (UM), community controls (CC).

In our cohort, rs867186-G variant and sEPCR levels were strongly associated in each disease group, with AG and GG genotypes having higher sEPCR levels than AA (Fig. 4). Children with SM who had genotype AG (n = 91, median, ng/ml [25^th^, 75^th^ percentile], 131 [107, 170]) had significantly higher levels than children with genotype AA (n = 390, 84.5 [65.7, 104], *P* < 0.001). Only three SM children had the GG genotype, and they had higher sEPCR level than the children with AA (n = 3, 194 [104, 211], *P* = 0.007) but not AG genotypes (*P* = 0.71, Fig. 4). Similarly, children with UM with the AG genotype (n = 13, 161 [142, 164]) had higher plasma sEPCR levels than those with AA (n = 25, 86.5 [75.4, 113], *P* < 0.001). The effect of rs867186-G variant was clearest in healthy CC children. Plasma sEPCR levels were higher with increasing presence of the G variant (AA (n = 79, 98.4 [87.8, 121]; AG, n = 25, 241 [203, 288]); GG, n = 6, 350 [319, 380], *P* < 0.006 for all comparisons, Fig. 4). The rs867186-G variant was similarly associated with sEPCR level at 6-months follow-up (Supplemental Figure 1).

**Figure 5:**
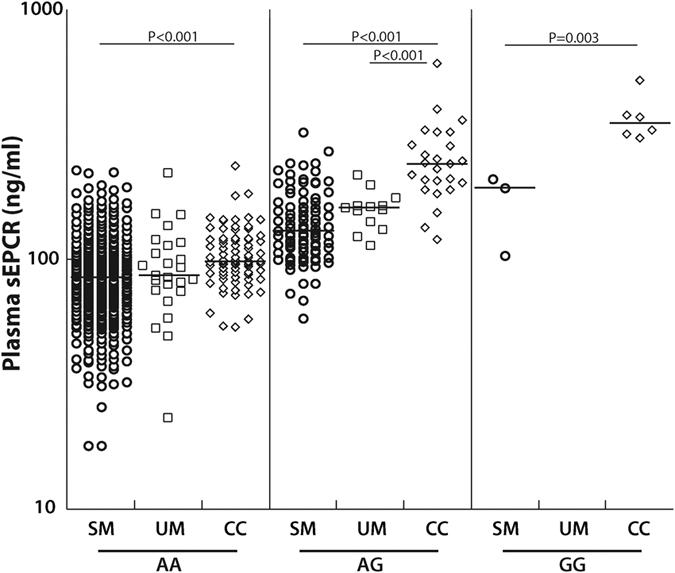
Plasma sEPCR levels are lower with increased disease severity when controlling for rs867186-G variant. For each genotype (AA, AG or GG) the median plasma sEPCR levels are represented for each group. The horizontal line represents median values. Severe malaria (SM), uncomplicated malaria (UM), community controls (CC).

For the AA, AG and GG genotypes, sEPCR levels were higher with decreasing disease severity (Fig. 5). Thus sEPCR levels were lower in children with severe malaria even after controlling for the rs867186-G variant.

### Relationships between inflammation, parasite biomass and endothelial activation and plasma sEPCR levels in children with severe malaria

Inflammation and parasite biomass can affect sEPCR levels, while EPCR can in turn affect endothelial activation. When comparing levels of markers of inflammation, endothelial activation and parasite biomass to sEPCR levels, all levels were log transformed (log base 10) because of their skewed distribution, so β-coefficients represent comparisons of log 10 increase in one factor to a log 10 increase in the other factor. After adjustment for age, plasma TNF-α levels correlated positively with sEPCR levels in children with severe malaria (β-coefficient 0.03, 95% CI 0.002–0.06, *P* = 0.04, Table 3). Plasma PfHRP-2 levels in the full study cohort had a negative but non-significant correlation with plasma sEPCR levels (β coefficient −0.01, 95% CI −0.03–0.007, *P* = 0.24). However, among children with severe malaria, sEPCR levels were positively associated with total (β-coefficient 0.05, 95%CI 0.03–0.08, *P* < 0.001) and sequestered parasite load (β-coefficient 0.04, 95% CI 0.02–0.07, *P* = 0.002, Table 3), after adjusting for age.

Among markers of endothelial activation, including von Willebrand Factor (VWF), angiopoietin 2 (Ang-2), intercellular adhesion molecule-1 (ICAM-1) and vascular cellular adhesion molecule-1 (VCAM-1), sEPCR levels were associated with increased levels of soluble ICAM-1 (β-coefficient 0.51, 95% CI 0.20–0.82, *P* = 0.001), but not with VWF, VCAM-1 and Ang-2 levels (Table 3).

### sEPCR levels in the cerebrospinal fluid of children with CM

**Figure 6:**
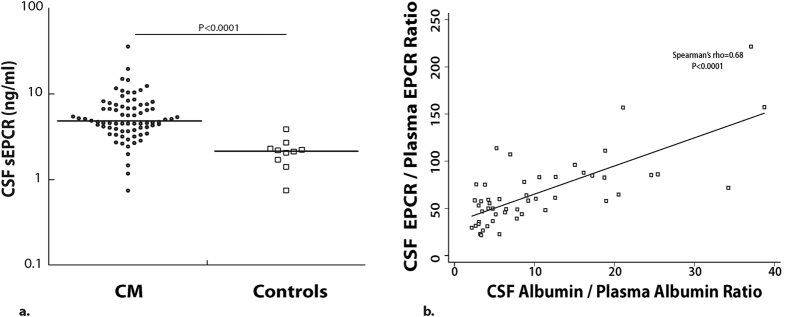
CSF sEPCR levels are elevated in children with cerebral malaria. (**a**) Levels of sEPCR were measured in CSF obtained in CM children who were in stable conditions for a spinal tap. Control CSF samples were obtained from asymptomatic children successfully treated for prior leukemia who had CSF obtained after treatment to rule out return of malignancy. (**b**) Spearman correlation of CSF-to-plasma albumin ratio vs. CSF to plasma sEPCR ratio for children with CM.

EPCR is also important in the central nervous system (CNS) as it transports aPC across the blood brain barrier (BBB)^40^ and facilitates neuroprotective effects of aPC^40^,^41^,^42^. Elevated levels of sEPCR in cerebrospinal fluid (CSF) could inhibit these neuroprotective effects by depleting available aPC. To assess the association of CSF sEPCR levels with adverse outcomes in CM, we quantified sEPCR in the CSF of children with CM. Median [25^th^ percentile, 75^th^ percentile] CSF sEPCR levels (ng/ml) were higher in children with CM (n = 76, 4.8 [3.9, 7.3]) than in control asymptomatic North American children with prior neoplastic disease (n = 10, 2.2 [1.8, 2.3], *P* < 0.0001, Fig. 6a). CSF sEPCR levels correlated positively with plasma sEPCR levels (Spearman’s rho = 0.34, *P* = 0.003) suggesting a passive diffusion due to BBB breakdown. To investigate this further, we assessed the association of CSF-to-plasma sEPCR ratio (CSF sEPCR×1000/Plasma sEPCR (ng/ml)) with CSF-to-plasma albumin ratio (CSF albumin×1000/Plasma albumin (mg/L)). The sEPCR ratio correlated positively with the albumin ratio (Spearman’s rho = 0.68, *P* < 0.0001, Fig. 6b), suggesting that the major source of sEPCR in the CSF of children with CM is transport from plasma across an impaired BBB.

### Association of plasma and CSF sEPCR with disease severity markers in cerebral malaria

Among cerebral malaria (CM) children that had plasma sEPCR quantified (n = 277), 30 died and of the children who survived, 80 children were discharged with neurologic deficits and 11 had neurologic deficits at 6-months follow-up. In children with CM, neither plasma nor CSF sEPCR was associated with mortality or neurologic deficits at discharge or 6-months follow-up (*P* > 0.10 for all, Table 4), adjusting for age. CSF and plasma sEPCR were also not associated with coma duration or seizure number during admission (*P* > 0.10 for all, Table 4). sEPCR levels were also not associated with neurocognitive outcomes (overall cognitive ability, associative memory, or attention) in children with CM under 5 years of age (*P* > 0.10 for all, Table 5).

Finally, we compared sEPCR levels in the children with CM who were malaria retinopathy positive versus negative. Children who were retinopathy positive had lower sEPCR levels, and difference approached statistical significance (n, median, ng/ml [25^th^ percentile, 75^th^ percentile] levels in retinopathy positive, n = 153, 88.7 [71.0, 115] vs. retinopathy negative, n = 72, 98.9 [72.8, 141], *P* = 0.07).

## Discussion

The present study found that in Ugandan children, the rs867186-GG genotype is more prevalent in healthy community children than in SM and is associated with increased sEPCR levels; that healthy community children have higher sEPCR levels than children with SM, and that among children with an initial episode of SM, those with repeated episodes of SM have lower sEPCR levels during the initial admission and at 6-month follow-up than those without repeated SM. Since sEPCR levels in other infectious and inflammatory processes are almost uniformly elevated, the present study’s findings suggest a distinctive role for sEPCR in severe malaria as compared to other infectious diseases, and support the idea that the rs867186-GG genotype might mediate protection from severe malaria through increased sEPCR levels.

The reduced prevalence of the rs867186-GG genotype in severe malaria is similar to the findings of a study of Thai adults^33^, but differs from studies in Ghanaian^34^ and Tanzanian children^35^, which found no association between the prevalence of rs867186-G variant and severe malaria^34^,^35^. In all these studies, rs867186-GG was uncommon, occurring in <5% of the population, suggesting that the benefits are either modest or counterbalanced by deleterious effects, such as the association of this variant with an increased risk of thrombotic disorders^43^. The inconsistencies between findings could arise from other genetic factors, diseases, or co-infections that differ between these study populations. Large multi-center studies including areas of differing malaria transmission are needed to understand the selection pressure, if any, on this gene and others involved in the aPC/EPCR system in Sub-Saharan Africa.

The present study also found that sEPCR levels were decreased in severe malaria, in contrast to the elevated sEPCR levels typically seen in other infections and disease processes characterized by inflammation. We did not see a significant difference in sEPCR levels between children with CM and SMA (data not shown). High sEPCR levels are seen in SLE^44^,^45^, before relapse in Wegener’s granulomatosis^46^, and in Behcet’s disease^47^. In sepsis, the findings are more nuanced, but the majority of the studies have shown elevated^44^,^48^,^49^ or similar^50^,^51^ levels of sEPCR in sepsis patients as compared to healthy individuals, with one study showing significantly lower sEPCR levels in patients with severe sepsis at the onset of organ failure than in healthy controls^52^. The differences in findings could be explained partially by the lack of rs867186-G genotyping, which is strongly associated with sEPCR levels. The present study’s findings on low sEPCR in SM are consistent with an earlier small study of children with severe malaria^35^, but contrast with a study of children from Benin in which sEPCR levels were higher in children with CM than in children with uncomplicated malaria, and in which the highest sEPCR levels were seen in children who died^36^. Differences in sample processing or testing, or differences in levels due to extremely severe disease in the Benin study (in which patients with CM had a 47% mortality rate) or differences in population genetics might have contributed to the differing findings in the Benin study. However, the present study, which has a sample size more than triple that of either previous study, clearly found that sEPCR levels are lower in severe malaria, and also showed that children readmitted with severe malaria had lower sEPCR levels than children not readmitted with severe malaria, further supporting an association of low sEPCR levels with severe malaria.

While the rs867186-G variant can affect the levels of sEPCR, we showed that even when controlling for the prevalence of this variant, children with SM had lower levels of sEPCR than CC (Fig. 5), suggesting that disease processes in SM are affecting the levels of sEPCR seen in SM. Why might plasma sEPCR levels be decreased in severe malaria? There are several potential reasons. Because sEPCR can bind to IEs^31^, the IE-bound EPCR may be cleared by the spleen or be removed during plasma processing. Binding of PfEMP-1 to EPCR could also provide an immune evasion mechanism for the parasite. Moxon *et al*. demonstrated that loss of EPCR was associated with parasite sequestration^32^, suggesting that interaction of IEs with EPCR may decrease detection of endothelial cell-bound EPCR. How this affects shedding of sEPCR is unknown; it is possible that IE binding to cell-bound EPCR could reduce EPCR shedding. Also, sEPCR could bind to activated neutrophils^53^, or due to its small size, leak into damaged organs as seen in the CSF of children with CM (Fig. 6b). Any or all of these processes could contribute to decreased systemic sEPCR in severe malaria. Determining the expression level of EPCR in subcutaneous tissues^32^ or circulating endothelial cells^45^ would complement our findings. Additionally, measuring sEPCR levels and parasite clearance at multiple time-points could help determine whether the changes in sEPCR are indeed due to a malaria-specific event.

In the present study, we found elevated levels of CSF sEPCR in CM children, similarly to a previous smaller study^32^, but unlike Moxon *et al*. we did not find strong evidence for local shedding of sEPCR since sEPCR and albumin ratios strongly correlated and there was no evidence of an upward shift in sEPCR ratios more than what would be predicted from a similar increase in albumin index (Fig. 6b). However, we could not measure albumin index in our control samples, and so could not rule out any local production of sEPCR. Furthermore, considering the nature of our study, we cannot determine causality and order of events. It could be that BBB leakage as a result of inflammation leads to increased sEPCR in the CSF, but it could also be that considering the cytoprotective effects of EPCR^25^, increased shedding of EPCR as a result of inflammation leads to loss of BBB integrity and increased leakage of plasma proteins including sEPCR. In our study, plasma and CSF sEPCR levels were not associated with mortality, morbidity (neurologic deficits, seizure number, coma duration), or cognitive outcomes in children with CM, suggesting that a further decrease in the already low sEPCR levels of children with severe disease did not lead to increased mortality or adverse neurologic complications. However, lower sEPCR levels at enrollment were associated with increased risk of readmission for malaria in children with severe malaria, suggesting that children with the lowest sEPCR levels during disease might have a greater risk of increased disease severity (requiring admission) with subsequent *P. falciparum* infection. This finding supports the idea that the ability to bind parasites with increased sEPCR might lead to protection from severe malaria, but the study numbers were small and additional studies are required to determine if this association is consistently seen.

Across all children, sEPCR levels had a non-significant but negative correlation with PfHRP-2 levels, as might be expected if increased parasite load led to increased binding of sEPCR in plasma. However, among children with CM or SMA, children with higher parasite biomass also had higher sEPCR levels. Since within disease groups, TNF-α correlated strongly with PfHRP-2 (Spearman’s rho 0.57, *P* < 0.0001), and TNF-α is known to be associated with severe disease^54^,^55^,^56^, it is possible that this correlation between sEPCR and PfHRP-2 reflects the second phase of a biphasic response: while initially EPCR binds IEs and sEPCR could be protective against sequestration, later in the disease stage, an increase in TNF-α levels in response to an increase in parasite biomass, leads to elevated shedding of sEPCR^27^. Moreover, considering the role of EPCR in endothelial stability we hypothesized that elevated levels of sEPCR would be associated with elevated endothelial activation in SM. When adjusting for TNF-α levels, sEPCR levels were associated only with elevated sICAM-1 (Table 3), emphasizing the multifactorial processes that could be contributing to endothelial activation in SM.

EPCR-binding PfEMP1 are large multi-domain proteins and are likely binding to other receptors. Therefore it will be important to determine the relative importance of other receptors working in concert with EPCR in severe malaria. *In vitro* studies and clinical studies across multiple research sites could provide much additional information on what induces production of sEPCR, how it is regulated and removed from the body, and how sEPCR levels relate to endothelial cell-bound EPCR.

In summary, our study found that in Ugandan children, the rs867186-GG genotype was associated with increased sEPCR levels and was less common in severe malaria, higher sEPCR levels were seen in healthy community children than in children with severe malaria, and lower sEPCR levels during severe malaria and in follow-up were associated with readmission for malaria. The findings suggest that sEPCR has a distinctive role in malaria, probably due to its binding to IEs. The mechanisms by which sEPCR levels are altered in severe malaria, the sequence of events, and the full consequences of decreased sEPCR levels are important areas for future studies.

## Materials and Methods

### Study design

This study was conducted at Mulago National Referral and Teaching Hospital in Kampala, Uganda between 2003–2013. Samples were obtained from two studies: the first study, during 2003–2005, enrolled children with cerebral malaria (CM), uncomplicated malaria (UM) and community controls (CC) between the ages of 3–12 years old, and the second study, during 2008–2013, enrolled children with CM, severe malarial anemia (SMA) and CC between the ages of 18 months to 12 years old. The overall goal of both prospective studies was to identify immunologic and genetic factors associated with neurologic and cognitive deficits in children with cerebral malaria under the working hypothesis that specific factors involved in the pathogenesis of CM and/or brain injury are associated with cognitive and neurologic deficits. The studies were reviewed and approved by the Ugandan National Council for Science and Technology (UNCST), the Makerere University School of Medicine Research and Ethics Committee, Case Western Reserve University and the University of Minnesota Institutional Review Board. Written informed consent was obtained from parents or guardians of study participants. The study methods were carried out in accordance with the approved guidelines.

Children between 18 months and 12 years of age, meeting the WHO definition for CM or SMA, were recruited from the Acute Care Unit at Mulago Hospital as previously described^1^. Cerebral malaria was defined as: 1) coma (Blantyre Coma Score [BCS] ≤2); 2) *Plasmodium falciparum* on blood smear; and 3) no other known cause of coma. Severe malarial anemia was defined as presence of *Plasmodium falciparum* on blood smear in children with hemoglobin <5 g/dL. Children with severe malaria were managed according to the Ugandan Ministry of Health treatment guidelines at the time, which included quinine treatment^1^. Children with UM (fever, *P. falciparum* on blood smear, no criteria for severe malaria, not admitted) were enrolled from the Mulago Hospital Acute Care clinic or from a Mulago Hospital outpatient malaria clinic.

Community children (CC) were recruited from the nuclear family, extended family or household compound area of children with CM, SMA or UM. Eligible CC were age 18 months to 12 years and currently healthy. Community children who were siblings of children with severe malaria were excluded from the present study analysis. Exclusion criteria for all children included: 1) known chronic illness requiring medical care; 2) known developmental delay; or 3) prior history of coma, head trauma, cerebral palsy, or hospitalization for malnutrition. Importantly, none of the community children were readmitted for severe malaria in the 6-month follow-up period, while 5.3% of the children with severe malaria were readmitted for severe malaria, demonstrating that the CC group did have protection against severe malaria as compared to the severe malaria group.

Neurologic examination was performed at discharge and six-months follow up^3^. Neurocognitive testing was performed among children <5 years in the second study at 12-month follow-up for overall cognitive ability, associative memory and attention, using the Mullen Scales of Early Learning, the Color Object Association Test and the Early Childhood Vigilance Test, respectively (CM, n = 139, SMA, n = 152, CC, n = 132), as previously described^1^. *PROCR* genotyping was done on samples with sufficient DNA quality and volume (551 SM (325 CM and 226 SMA), 71 UM, 172 CC). Plasma sEPCR levels were tested in children at baseline and 6-month follow-up if a sufficient volume was collected (Fig. 1). Cerebrospinal fluid (CSF) sEPCR levels were measured in CM children who had adequate CSF volume for testing (n = 76). Control CSF samples were obtained from asymptomatic children successfully treated for prior leukemia who had CSF obtained after treatment to rule out return of malignancy (ruled out in all).

### DNA extraction and *PROCR* rs867186 genotyping

Genomic DNA was isolated from whole blood samples of severe malaria patients using the DNeasy Blood and tissue kit (Qiagen, Valencia, CA) and from filter papers for UM patients and CC using QIAamp 96 DNA Blood Kit (Qiagen, Valencia, CA). E4F (5′- GCTTCAGTCAGTTGGTAAAC-3′) and E4R (5′- TCTGGCTTCACAGTGAGCTG-3′)^37^ were used to amplify the region of the *PROCR* gene containing rs867186 and rs9574. Genotyping of rs867186 and rs9574 was done by initially amplifying the region of interest using HotStar Taq plus master mix (Qiagen, Valencia, CA), followed by Sanger Sequencing (ABI 3730xl, University of Minnesota Genomics Center).

### Laboratory testing

Soluble EPCR in plasma, serum and CSF were quantified using Asserachrom^®^ sEPCR immunoassay (Stago, France). Plasma and serum were diluted according to manufacturer’s instructions (1:51); CSF was diluted 1:2. The Asserachrom^®^ sEPCR immunoassay uses antibodies directed against the PC binding site of sEPCR.

Peripheral blood smears were assessed for *Plasmodium* species by microscopy with Giemsa staining using standard protocols. PfHRP-2 quantification was performed using the Malaria Ag CELISA (Cellabs, Brookvale, Australia). Sequestered parasite biomass was calculated as previously described^57^. Plasma soluble intercellular adhesion molecule-1 (sICAM-1), vascular cellular adhesion molecule-1 (sVCAM-1), and TNF-α levels were measured by magnetic cytometric bead assay (R&D Systems, Minneapolis, MN and EMD-Millipore, Billerica, MA, respectively) according to manufacturer’s instructions with a BioPlex-200 system (Bio-Rad, Hercules, CA). Plasma angiopoietin-2 (Ang-2) and von Willebrand Factor (VWF) levels were quantified using the human angiopoietin 2 DUO ELISA kit (R&D Systems, Minneapolis, MN) and REAADS von Willebrand Factor activity ELISA kit (Corgenix, Broomfield, CO), respectively. Plasma and CSF albumin were quantified by the Advanced Research and Diagnostic Laboratory at the University of Minnesota.

### Statistical analysis

Fisher’s exact test for 2 × 2 tables was used to compare prevalence of *PROCR* variants between the control and malaria groups, when considering a dominant or recessive model. Fisher’s exact test for 2 × 3 tables was used for the additive model. To control for multiple comparisons, only *P* < 0.008 was considered statistically significant in both tests.

Measures with skewed distributions, including sEPCR levels, were replaced by their common logs (log to base 10) for ANOVA or regression analyses. sEPCR levels were compared between groups or between genotypes using ANOVA, followed by Tukey’s post-hoc test to control for multiple comparisons. Clinical and laboratory findings for children in the different disease groups were compared using the chi-squared test if categorical and if continuous, ANOVA followed by Tukey’s post-hoc test. Regression analyses used linear regression for continuous outcomes and logistic regression for categorical outcomes. All regression analyses were adjusted for age.

## Additional Information

**How to cite this article**: Shabani, E. *et al*. The endothelial protein C receptor rs867186-GG genotype is associated with increased soluble EPCR and could mediate protection against severe malaria. *Sci. Rep.* **6**, 27084; doi: 10.1038/srep27084 (2016).

## Supplementary Material

Supplementary Information

## Figures and Tables

**Table 1:** Age and sex of children with severe or uncomplicated malaria and community children.

	Severe Malaria (SM, n = 551)	Uncomplicated Malaria (UM, n = 71)	Community Children (CC, n = 172)	P^a^
Age (months) median (IQR)	41.7 (28.1–59.3)	78.0 (58.6–108)	55.5 (36.1–84.9)	<0.0001^b^
Sex, male n (%)	329 (59.7)	31 (43.7)	85 (49.4)	0.005^c^
*P. falciparum* peripheral blood density (parasites/μl)^e^, median (IQR)	39660 (9900–191380)	33420 (7860–116580)	0 (0–0)	<0.0001^d^

^a^ANOVA, Tukey post-hoc test adjustment for multiple comparisons with log10 transformed values for variables with no normal distribution. Chi-squared test was used for categorical variables, with *P* < 0.017 considered significant to control for multiple comparisons.

^b^In post-hoc testing, all pairs of groups differ significantly.

^c^SM significantly different from UM.

^d^In post-hoc testing, CC differ from SM and UM.

^e^n = 540 for SM, n = 69 for UM and n = 131 for CC.

**Table 2:** Prevalence of rs867186-G variant in malaria disease groups and community children.

	rs867186 (A4600G)			P^a^ Additive model	P^a^ Recessive model	P^a^ Dominant model
	AA, N (%)	AG, N (%)	GG, N (%)		GG vs. AG + AA	GG + AG vs. AA
SM (N = 551)	446 (80.9)	102 (18.5)	3 (0.6)	**0.006**^b^	**0.002**^b^	0.38^b^
UM (N = 71)	57 (80.3)	14 (19.7)	0 (0)	0.28^c^	0.11^c^	0.73^c^
CC (N = 172)	134 (77.9)	31 (18.0)	7 (4.1)	Reference	Reference	Reference

SM, severe malaria (cerebral malaria or severe malarial anemia); UM, uncomplicated malaria; CC, community children.

^a^Fisher’s exact test is used. *P* < 0.008 considered significant to control for multiple comparisons.

^b^SM vs. CC.

^c^UM vs. CC.

**Table 3:** Association of plasma sEPCR levels with endothelial activation markers and PfHRP-2 levels in children with severe malaria.

	TNF-α (pg/ml)		PfHRP-2 (ng/ml)		Sequestered biomass		VWF (% of normal)		Ang-2 (ng/ml)		sICAM-1 (ng/ml)		sVCAM-1 (ng/ml)	
	β^a^ coefficient(95% CI)	P	β^a^ coefficient(95% CI)	P	β^a^ coefficient(95% CI)	P	β^b^ coefficient(95% CI)	P	β^b^ coefficient(95% CI)	P	β^b^ coefficient(95% CI)	P	β^b^ coefficient(95% CI)	P
Plasma sEPCR (ng/ml)	0.03 (0.002–0.06)	0.04	0.05 (0.03–0.08)	<0.001	0.04 (0.02–0.07)	0.002	0.11 (−0.10–0.33)	0.29	0.08 (−0.22–0.38)	0.60	0.51 (0.20–0.82)	0.001	0.07 (−0.06–0.20)	0.31

All values were log-transformed (log10).

^a^Models adjusted for age.

^b^Models adjusted for age, and systemic TNF-α levels.

**Table 4:** Relationship of plasma and CSF sEPCR levels to mortality and neurologic morbidity in children with cerebral malaria.

	Mortality		Neurologic deficit (discharge)		Neurologic deficit (6mo)		Number of seizures after admission		Coma duration (hours)	
	OR (95% CI), n	P	OR (95% CI), n	P	OR (95% CI), n	P	β coefficient (95% CI), n	P	β coefficient (95% CI), n	P
Plasma sEPCR (ng/ml)	3.13 (0.36–27.39)^a^,n = 277	0.30	3.53 (0.76–16.35)^b^,n = 243	0.11	3.87 (0.12–128)^c^,n = 233	0.45	0.24 (−0.05–0.54),n = 141	0.11	0.01 (−0.25–0.28),n = 242	0.92
CSF sEPCR (ng/ml)	4.30 (0.17–110)^d^,n = 76	0.38	0.99 (0.13–7.65)^e^,n = 70	0.99	0.09 (0.0005–16.14)^f^,n = 67	0.37	0.20 (−0.13–0.53),n = 38	0.23	0.02 (−0.37–0.40),n = 70	0.92

All models were adjusted for age. Plasma and CSF sEPCR levels were log transformed (log 10).

^a^Survived (n = 247), died (n = 30).

^b^Discharged with neurologic deficits (n = 80) vs. without (n = 163).

^c^Neurologic deficits at 6-months follow-up (n = 11) vs. not (n = 222).

^d^Survived (n = 70), died (n = 6).

^e^Discharged with neurologic deficits (n = 26) vs. without (n = 44).

^f^Neurologic deficits at 6-months follow-up (n = 4) vs. not (n = 63).

**Table 5:** Relationship of plasma and CSF sEPCR levels with cognitive outcomes at 12 months follow-up in children with cerebral malaria.

	Overall cognition		Associative memory		Attention	
	β coefficient (95% CI), n	P	β coefficient (95% CI), n	P	β coefficient (95% CI), n	P
Plasma sEPCR (ng/ml)	−1.26 (−2.90–0.38). n = 120	0.13	−0.65 (−1.48–0.18), n = 120	0.12	−0.82 (−1.83–0.18), n = 123	0.11
CSF sEPCR (ng/ml)	−0.45 (−3.18–2.28), n = 47	0.74	−0.33 (−0.99–0.33), n = 47	0.31	1.09 (−0.24–2.41), n = 47	0.11

All models were adjusted for age. Plasma and CSF sEPCR log transformed (log 10).
