# Supplementary material for: The endothelial protein C receptor rs867186-GG genotype is associated with increased soluble EPCR and could mediate protection against severe malaria
Source: Sci Rep. 2016 Jun 3;6:27084. doi: 10.1038/srep27084 (PMC4891778; doi:10.1038/srep27084)

The endothelial protein C receptor rs867186-GG genotype is associated with increased soluble EPCR and could mediate protection against severe malaria

Estela Shabani, Robert O. Opoka, Paul Bangirana, Gregory S. Park, Gregory M. Vercellotti, Weihua Guan, James S. Hodges, Thomas Lavstsen and Chandy C. John

	rs9574 (G4678C)			P[a] Additive model	P[a] Recessive model	P[a] Dominant model
	GG, N (%)	GC, N (%)	CC, N (%)		CC vs. GC+GG	GG vs. GC+CC
**SM (N=550)**	382 (69.5)	155 (28.2)	13 (2.3)	0.14[b]	0.28[b]	0.29[b]
**UM (N=71)**	59 (83.1)	11 (15.5)	1 (1.4)	0.32[c]	0.44[c]	0.18[c]
**CC (N=170)**	126 (74.1)	37 (21.8)	7 (4.1)	Reference	Reference	Reference

Supplemental Table 1. Prevalence of rs9574-C variant in malaria disease groups and community control

SM, severe malaria (cerebral malaria or severe malarial anemia); UM, uncomplicated malaria ; CC, community children

[a]Fisher's exact test used. *P*<0.008 considered significant to control for multiple comparisons

[b]SM vs. CC

[c]UM vs. CC

	H1/H3, N (%)	H1/Hx or H3/Hx, N (%)	Hx/Hx, N (%)	P[a] (H1/H3)
**SM (N=550)**	21 (3.8)	231 (42.0)	298 (54.2)	0.99[b]
**UM (N=71)**	2 (2.8)	22 (31.0)	47 (66.2)	0.99[c]
**CC (N=170)**	6 (3.5)	69 (40.6)	95 (55.9)	Reference

Supplemental Table 2. Prevalence of rs9574-C and rs867186-G variants in malaria disease groups and community control

SM, severe malaria (cerebral malaria or severe malarial anemia); UM, uncomplicated malaria ; CC, community children

[a] Fisher's exact test used. P<0.025 considered significant to control for multiple comparisons; H1/H3 having at least one copy of each variant (heterozygous or homozygous for H1 and heterozygous or homozygous for H3)

[b]SM vs. CC

[c]UM vs. CC

Supplemental Figure 1. rs867186-G is associated with increased levels of systemic sEPCR at 6-month follow-up. sEPCR levels are represented on a logarithmic scale and each disease group is separated by rs867186 genotype: AA, AG or GG. The horizontal line represents median values. Severe malaria (SM), community controls (CC).

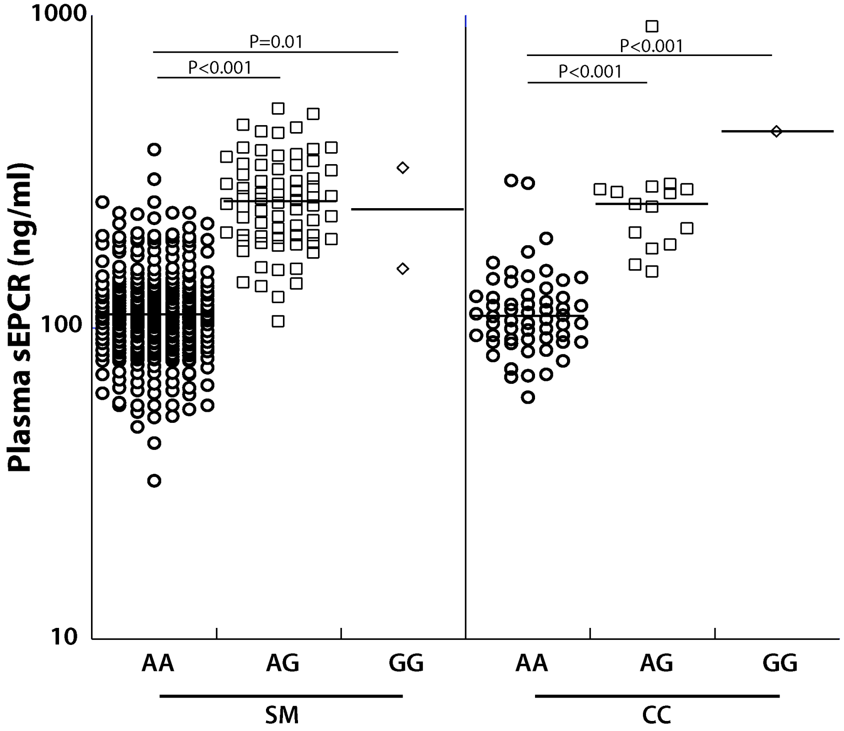

Supplement: Supplementary Information [file srep27084-s1.pdf]
